# Supplementary material for: Prevalence of visual impairment and its causes in adults aged 50 years and older: Estimates from the National Eye Surveys in Malaysia
Source: PLoS One. 2024 Oct 24;19(10):e0299768. doi: 10.1371/journal.pone.0299768 (PMC11500914; doi:10.1371/journal.pone.0299768)
Supplement: Attachment — Submitted filename: Response to reviewers.pdf [file pone.0299768.s002.pdf]

PLOS ONE Journal

Manuscript Title: Prevalence of visual impairment and its causes in adults aged 50 years and older: Estimates from the National Eye Surveys in Malaysia

Dear Srinivas Marmamula,
Academic Editor
PLOS ONE

We would like to extend our sincere appreciation to the editor and reviewers for their insightful comments and valuable suggestions on our manuscript. We have modified the manuscript accordingly, and the detailed corrections are listed below point by point:

Reviewer #1
This is an important paper and the data will provide useful feedback to eye service organizers in Malaysia. There are three items that need to be addressed as listed here:

Comment 1:

Ethical approval information is listed in the initial application, but is not included in the report narrative itself. Please include the ethical approval information in the report narrative in the Methods section per journal requirements and as requested on the submission form (page. 5 of PDF package).

The ethical approval information has been added to the 'Methods' section, highlighted in red fonts, and incorporated into the manuscript.

Line 219-222:

Ethics approval
Medical Research and Ethics Committee of the Malaysian Ministry of Health (Research ID NMRR-19-197-46172). The study was conducted in accordance with the tenets of the Declaration of Helsinki.

Comment 2:

Inter-observer agreement information is found in three separate parts of the manuscript making it easy for the reader to gloss over this information and miss something important. You mention inter-observer agreement testing in the methodology (lines 106-110) and later list kappa values as being poor, fair, moderate, good or very good in a Definitions section (lines 152-153) and under the results section (lines 184-187) you provide a breakdown of inter-observer agreement scores by percent for fair, moderate, good and very good.

Please note that the typical convention is to discuss inter-observer agreement in the methodology section, including the Kappa statistic results. This is part of the preparation, thus methodology. Please also note that the RAAB manual lists Kappa statistic of 0.60 or greater as being acceptable. I do not understand what

you mean when you provide a percentage breakdown. The way this section is written (lines 184-187) is appears that some of the results are below what is considered acceptable. If results are below what is acceptable (Kappa 0.60 or greater) were teams further trained and re-tested? If not, this would be a serious limitation that would also need to be discussed in a limitations section.

Please consolidate inter-observer agreement information in the methodology section.

Please state clearly if the results were acceptable per RAAB standards. If results were not acceptable, please explain. If results were not acceptable also discuss the reasons and implications for this in the Limitations section.

We acknowledge the importance of consolidating this information in the methodology section, as per the typical convention.

We have revised the manuscript. The assessment of inter-observer variation (IOV), the Kappa coefficient values and the results of IOV have been included in the 'Survey Method' section (under subheading 'training'), highlighted in red fonts. (Line 130-140)

We have recalculated the values and presented them as ≤0.60 or >0.60. Kappa Tables for both Sarawak and the Eastern Region are also attached for your further perusal and decision whether they should be included in the main manuscript.

Comment 3:

Limitations - there is no section in the discussion for limitations (or strengths and limitations) of this study. This is a must. Your mention of the convenience of using the RAAB software package and digital data entry, etc. (lines 246-252) can be shortened and included as a strength. Please read several past RAAB publications for examples of what to include in the strengths and limitations section. Problems with inter-observer agreement not being up to standard should be discussed here as a limitation.

The limitation section has been added in the main text, highlighted in red fonts.

Line (201-218):

Limitation

The doctors in the survey team (responsible for diagnosing ocular conditions and delivering medical explanation to the subjects when required) were the department's youngest medical doctors or ophthalmology trainees. They have varying ophthalmology experience and exposure to basic eye care training. Hence, there were disagreements during the inter-observation assessment. Given the long duration of fieldwork, most hospitals in both regions could not allow more senior doctors to be recruited because of workforce shortage.

Data collection coincided with the pre-election time for the state legislative assemblies in both regions. It was impossible to reschedule the survey activities because the election date was only announced after all the logistics planning for the

fieldwork, including the workforce mobilization, had been done. Although the highest level of permission to visit the houses, examine, and interview the subjects was applied and approved by the local authorities, there was resistance from certain subjects/communities to the examination/interview, alleging that the study had political intentions. The data collectors took more time to explain to the community leaders and subjects that this study had no political agenda. In several Enumeration Blocks (EBs), subjects had to be revisited before they agreed to participate. It resulted in a prolonged stay for the data collectors in the EB; hence, more money was spent on their accommodation and other logistics.

Other comments

Smaller items include:

Comment 1:

Line 264-265 - you mention the prevalence of Vision impairment in your study is lower than other countries in the region and provide citations, but you don't actually provide any numbers for comparison. It would be useful to note what the range of Vision impairment is among the studies you are citing.

Line 307-311:

In a global comparison of the prevalence of blindness among countries using the same RAAB methodology, Malaysia reported a lower prevalence in 2023, with the Sarawak zone at 0.6% and the Eastern zone at 0.8%. In contrast, Indonesia's Jawa Barat had a prevalence of 2.8% in 2014, Indonesia's Jawa Timur at 4.4%, Jawa Tengah at 2.7%, and Viet Nam Dinh at 2.1% in 2015 [21].

Ref [21]: Rapid Assessment of Avoidable Blindness: Survey Data. Available at: https://www.raab.world/survey-data

Comment 2:

Conclusion - The first sentence "The prevalence of blindness and severe VI were reduced" is vague. I would make this sentence much more informative. For example "The prevalence of blindness and Vision impairment was lower in 2023 compared to 2014 in both the Sarawak region and the Eastern region."

This comment has been addressed in the manuscript, highlighted in red fonts.

Line 400-406: The prevalence of blindness and vision impairment was lower in 2023 compared to 2014 in both Sarawak and the Eastern Region of Malaysia.

Comment 3:

Line 109 - this would be more clear if you stated "..and a test cluster in one of the nearby EB's during fieldwork."

This comment has been addressed in the manuscript, highlighted in red fonts. (Line 117-119)

Comment 4:

Lines 137-139 you state. "Should subjects have a visual impairment, the primary cause was identified........." Was the diagnosis of primary cause of vision impairment made via RAAB / WHO protocols? if so you should state this. If not, you should note why.

Line 177-179: Should subjects have a visual impairment, the primary cause was identified (following the RAAB protocol), and the subjects were referred to the nearest ophthalmic care facility for further management.

Comment 5:

Lines 141-143. I did not understand what you mean when you state "......six survey teams were assess in their agreements asking 15 questions to subjects comprising patients and staff......" What questions? I don't understand this sentence.

For the Inter-observer Variation (IOV), the agreement was evaluated based on 15 questions related to vision and eye health assessments. These questions encompass aspects such as the use of spectacles for distance and near vision, acuity measurements for distance vision (presenting, corrected, pinhole, uncorrected), lens status for both eyes, and causes of poor vision for the right and left eyes.

Table 1 and Table 2 present the summary of Kappa Values comparing the survey team to the gold standard team during the IOV assessment for RAAB in Sarawak and the Eastern Zone. Both tables will not be included in the main manuscript but will be submitted as ‘other’ for reference.

Comment 6:

Lines 176-177 you mention double data entry. As I recall, the standard RAAB survey package and use of tablets do not use double data entry.

Inconsistency check and double data entry were the manual features of RAAB6. What we meant by the statement was, RAAB7 includes this capability in the software automatically, therefore enhancing the quality and integrity of the collected data. We apologize, we decided to omit this statement to avoid confusion.

Reviewer #2

This is a good article, in the sense that it reports prevalence of blindness and visual impairment after an intervention which followed a baseline study done in 2014. However, there a few points to consider:

Comment 1:

The term visual impairment, though not controversial in itself, should be distinguished from "blindness", in that the categories is defined in terms of Visual impairment. Hence, when referring to all vision loss categories, it is better to say "blindness and / or visual impairment".

This comment has been addressed in the main text, highlighted in red fonts.

Comment 2:

There are a few typographical, grammatical or semantic errors that can be corrected, as suggested below:

Comment:
Line 33: visual acuity "test" in stead of "check"

This comment has been addressed in the main text, highlighted in red fonts.

Line 32-35:

The prevalence of blindness and/or visual impairment (blindness, severe, moderate, and early) and its primary cause were determined through a visual acuity test and eye examination with a hand-held ophthalmoscope.

Comment:
Line 76: "aimed" in stead of "aims"

This comment has been addressed in the main text, highlighted in red fonts.

Line 86-87:

This study aimed to determine the difference or improvement in the prevalence of blindness and/or visual impairment between the two administrative regions from the previous survey.

Comment:
Line 103: "Selected" instead of "Individual"

This comment has been addressed in the main text, highlighted in red fonts.

Line 111-112:

Selected EB codes and the corresponding maps were then used to identify the location of the EBs during fieldwork data collection.

Comment:
Line 107: "Western Pacific" can be omitted

This comment has been addressed in the main text

Line 115-116:

Those training sessions by a certified RAAB trainer were to ensure data quality and strict adherence to study protocol.

Comment:
Line 109: "actual" can be replaced with "pilot"

This comment has been addressed in the main text, highlighted in red fonts.

Line 117-119:

Survey team members were required to attend four training days as preparation, including RAAB lectures, inter-observer variation assessment and a pilot survey in a test cluster in one of the nearby EB's during fieldwork.

Comment:
Line 123: It should be made clear that the data so recorded will not be included in the prevalence and causes analysis

This comment has been addressed in the main text, highlighted in red fonts.

Line 152-154:

If the subject could not be examined after three revisits, this person would be recorded as `Not Available' and included in prevalence and cause analysis.

Comment:
Line 125: Ordinarily, this should have meant a resident of that cluster or region, as non-resident participants will introduce bias.

This comment has been addressed in the main text, highlighted in red fonts.

Line 155-157:

Subjects were recruited if they were 50 years and older, residing in the area for at least six months, and provided informed consent. Non-residents were excluded from the study.

Comment:
Line 136: If this refers to Peek VA, it should be stated and appropriately referenced.

Line 166-169:

It was followed by visual acuity assessment conducted at a distance of three meters using tablets installed with the RAAB7 application which incorporates built-in quality control measures through the PEEK Vision platform.

Ref [9]: Rapid Assessment of Avoidable Blindness: About RAAB7. Available from: https://www.raab.world/about-raab/raab7

Comment:
Line 155: There should be explanation why "best correction" is not used as measurement.

Line 186-189:

The best correction would require refraction, which would be time-consuming and impossible in the field without proper equipment and expertise. It was also not measured because it would deviate from RAAB protocol, which only measured corrected VA (using a pinhole).

Comment:
Line 190: The sentence starting with "These subjects..." can be omitted.

This sentence has been removed from the main text.

Comment:
Line 246: NES III should be written in full at first mention in discussion.

This comment has been addressed in the main text, highlighted in red fonts.

Line 286-287:

The availability of RAAB7 software facilitated data entry and cleaning during National Eye Survey III (NES III).

Comment:
Line 262: Insert "surveys" after RAAB.

This comment has been addressed in the main text, highlighted in red fonts.

Line 303-304:

The prevalence of blindness during the six simultaneous RAAB survey (NES II 2014) in Malaysia ranged from 0.5-1.9% (with a calculated weightage of 1.2%) [5].

Comment:

Line 265: This is unclear, but can be improved by relating it to the context suggested in point 3.

This comment has been addressed in the main text, highlighted in red fonts. The discussion will be discussed in point 3.

Comment:
Line 274: "strengthened" can be replaced with "modified to incorporate"

This comment has been addressed in the main text, highlighted in red fonts.

Line 357:

One of the national initiatives which can potentially be modified to incorporate for diabetic retinopathy screening is the *Klinik Katarak-Kementerian Kesihatan Malaysia* (Cataract Clinic Ministry of Health Malaysia, *KK-KKM*), an outreach arm of the ministry to reach the population (Fig 3).

Comment:

Line 280: The sentence can be modified as follows: "The KK-KKM" uses buses to transport..."

This comment has been addressed in the main text, highlighted in red fonts.

Line 363:

The KK-KKM uses buses to transport surgical and medical equipment along the selected service routes according to the location of Provincial Hospitals, which are used as the primary service sites.

Comment 3:

There is a need to provide context to the situation in the two regions of Malaysia, in order to make the article more palatable for a more regional and global audience. I would recommend one or two paragraphs (after the first paragraph of the Introduction) describing the current situation of blindness and visual impairment, first regionally (western Pacific), then Malaysia as a country, then the two study regions. This paragraph should summarise the epidemiology and service delivery aspects at the time of the study implementation.

Thank you for your invaluable feedback and insightful suggestions. This comment has been addressed in the main text, highlighted in red fonts.

Line 63-71

In 2010, over 90 million people in the Western Pacific Region were visually impaired, including more than 10 million blind. Blindness or visual impairment can be avoided in 80% of cases with appropriate treatment or early prevention [4]. In Malaysia, the estimated prevalence of blindness in the country from findings of the Six Simultaneous National Eye Survey, NES II (2014) was 1.2%. Untreated cataracts were the leading cause, followed by diabetic retinopathy and glaucoma. When estimated by individual PBL administrative regions (Fig 1), there were discrepancies between the regions: Northern (1.5%), Eastern (1.4%), Central (0.5%), Southern (0.9%), Sabah (1.9%) and Sarawak (1.6%) with untreated cataract remained the main cause of blindness across the regions [5].

One of the national action plans planned and implemented after the survey was introducing a community eye care program, which could potentially reach the underserved population, especially in remote areas. Eastern Region and Sarawak were selected as the regions where this program would be piloted because they had the necessary eyecare ownership and network among the hospitals, the health state departments, the NGOs and the community leaders, which would ensure the

sustainability of a community program. Each region had a Public Health Ophthalmologist who could monitor and champion the program.

Ref [4]: Donatella P, Silvio PM. Global estimates of visual impairment: 2010. Br J Ophthalmol. 2012;96(5):614-618. DOI: 10.1136/bjophthalmol-2011-300539 PMID: 22133988

Ref [5]: Chew FLM, Salowi MA, Mustari Z, Husni MA, Hussein E, Adnan TH, et al. Estimates of Visual Impairment and its Causes from the National Eye Survey in Malaysia (NESII). *PLoS One*. 2018;13(6):e0198799. DOI: 10.1371/journal.pone.0198799 PMID: 29944677

Comment 4:

The author must confirm which of the WHO or IAPB "owns' the RAAB protocol, and reference it appropriately (Line 81).

The Rapid Assessment of Avoidable Blindness (RAAB) protocol is developed and owned by the International Center of Eye Health, London School of Hygiene and Tropical Medicine.

The word WHO recommended has been added, highlighted in red fonts, and incorporated into the manuscript.

Line 91-92:

These cross-sectional, population-based surveys, which followed the World Health Organization (WHO)-recommended RAAB protocol, were conducted simultaneously from 27 July 2023 to 7 October 2023.

Comment 5:
The training should summarise the main techniques covered in the training.

The main techniques covered in the training have been addressed in the manuscript and are highlighted in red fonts under the subheading "Training." on line 113-128

Comment 6:
Elaboration explaining the fundus examination after dilatation should be included.

This comment has been addressed in the main text, highlighted in red fonts.

Line 171-176:

The fundus examination was conducted using a direct ophthalmoscope after pupil dilatation with dilating eye drops, especially in cases where vision is poor (<6/12). The aim of the fundus examination was to look for abnormalities in the posterior segment of the eye which could potentially be the primary cause of visual impairment (main cause of presenting VA<6/12). The data collectors were trained to look for

signs of myopic degeneration, glaucoma, diabetic retinopathy or age related macular degeneration.

Comment 7:

The section (starting from line 140) describing the IOV test should be part of the Training section.

The section describing the IOV test has been relocated under the Training section as per the feedback. This adjustment has been highlighted in red fonts in the main text on line 130-140.

Comment 8:

The NES definitions starting at line 148 should be incorporated into the paragraph which starts at line 62.

The NES definitions have been integrated into the 'Introduction' section as per the feedback provided. This revision has been addressed in the main text, highlighted in red fonts.

Comment 9:
The section starting from line 152 should be incorporated into the paragraph about the IOV test (see 6 above).

This comment has been addressed in the main text, highlighted in red fonts.

Comment 10:

The IOV test is an internal quality control measure. It may or may not be considered as a study finding.

The IOV result has been removed from the Result section and submitted as 'Other' in the manuscript submission.

No comment no 11

Comment 12:
The explanation for greater representation of females could surmise other reasons from literature.

The explanation for the greater representation of females has been incorporated into the manuscript, highlighted in red fonts.

Line 295-298:

The greater representation of females in the survey could be influenced by traditional gender roles in Malaysia, where females are available because they are likely to stay at home taking care of the family's daily needs throughout the day while the males go out to work.

Comment 13:
The explanation for lesser representation of participants aged under 60 years could surmise other reasons from literature.

Similar to our response to comment no 12,

Line 300-302:

The younger individuals could have competing priorities, such as work commitments outside the house when the survey was done.

Comment 14:

It should be noted that the NES III prevalence of moderate visual impairment in both regions are higher than those in NES II, with possible explanation.

Similar answer to comment 15 and 16

Comment 15:

There should be reference made to the cause-specific changes over the two survey periods.

Response will be given based on the three main causes.

Line 319-325:

There was an added workflow to check visual acuity in NESIII (refer to definition in methodology). This workflow was intended to help calculate the effective Refractive Error Coverage (eREC) [22]. In NESII, either PVA (with available correction) or PinVA (with available correction) were tested. The increasing percentage of untreated refractive error was probably due to the increasing detection of refractive errors due to under/overcorrections or improper glasses fitting. Whether it was due to a rising prevalence of myopia among people 50 years and older, further research with proper refraction in the field needs to be done.

Line 336-350:

In case of cataract, when comparing NES II to NES III, although the prevalence of blindness was lower in both regions during NESIII, there was an increase in the percentage of untreated cataract as the cause of blindness in both regions and severe VI in the Eastern Region. The demographic population could partially explain these changes, i.e., the increased percentage of each age group 50 years and older (except for the 50-59 age group in the Eastern Region). The other possible explanation was that the amount of awareness and advocacy from the eye care delivery was still inadequate among the cataract population in the blind category.The percentages of severe, moderate and mild VI appeared to reduce in Sarawak. The percentages of moderate and mild VI also appeared to decrease in the Eastern Region.

In general, diabetic retinopathy as the cause for blindness and VI appeared to decrease except for moderate and mild VI in Sarawak. This improvement could be attributed to the national priorities and policies on Diabetes Mellitus, not KK-KKM initiatives [23]. The increase in the percentage of DR as the cause for moderate and mild VI in Sarawak could be associated with geographical access or coverage in DR screening within the state. Further research and analysis are needed as the issue is beyond the scope of this study.

Comment 16:

It should be noted that refractive error is second most prevalent cause of moderate visual impairment in both regions, with possible explanations.

Possible explanations for this finding have been provided and integrated into the main text, highlighted in red fonts.

Line 314-318:

Untreated refractive error as the second most common cause of moderate visual impairment in both regions could be attributed to various factors. Limited access to corrective eyewear, lack of awareness about refractive errors, and challenges in obtaining regular eye examinations may collectively contribute to the impact of refractive error on visual impairment.

Comment 17:

The sections starting at line 287 and 295 are too expansive, should be omitted, but can be referred to as a link or appendix in the preceding paragraph.

We have summarized the paragraph and has been incorporated in the main text, highlighted in red fonts.

Line 370-375:

The service concept revolved around providing eye care services near patients' homes and optimizing existing facilities in provincial hospitals for cataract surgeries instead of operating in mobile units [25-27]. The KK-KKM project in both regions emphasized scheduled trips for screening, surgeries, and follow-up visits after one month to evaluate visual outcomes. A fixed timetable was distributed to all provincial hospitals at the start of the year, enabling individuals in remote areas to plan their visits for eye treatment effectively.

Comment 18:

Explanation should be proffered for the low prevalence of other avoidable causes of blindness and visual impairment, and why cataract is still the most common cause.

The explanation for the low prevalence of other avoidable causes of blindness and visual impairment, as well as why cataract remains the most common cause, has been included in the manuscript. This addition has been highlighted in red fonts for reference.

Line 326-335:

The possible reason on why cataract remained the most common causes of blindness and vision impairment was due to the survey protocol. The specific cause of poor

vision (worse than 6/12) would be captured for individual eyes. However, if the cause was different between the eyes, the principal (main) cause of poor vision would be selected. The principal cause of poor vision was the condition more easily treated (to move the person from having VI to having normal vision). For example, in between diabetic retinopathy vs cataract, the principal cause of poor vision was cataract. The other possibility was because of the high percentage of untreated cataract in the blind category, the absence of fundus view did not allow for the detection of other avoidable blindness in the eye/person, hence the low prevalence of other avoidable blindness or VI.

Comment 19:
A postulate should be presented to suggest what impact could be anticipated from the surveys of the other 4 regions.

We have summarized the paragraph and has been incorporated in the main text, highlighted in red fonts.

Line 389-396:

Although implemented in Sarawak and the Eastern Region only, KK-KKM work concept was emulated by other regions. Many states started expanding their cataract service as outreach initiatives, not using dedicated mobile units but different methods, such as transporting patients to the main hospital or equipment to provincial hospitals using other smaller vehicles. The financial allocation, priorities, policies, and strategic plan for all cataract surgical services are under a single common governance (MOH). We postulate that positive results would also be seen in the other four regions if we ran the surveys concurrently.

Comment 20:

The conclusion should emphasize the gains made as evidence of this study, but also propose a way forward for eye care programming in Malaysia, based on the Discussion.

Line 400-406:

The prevalence of blindness and vision impairment was lower in 2023 compared to 2014 in both Sarawak and the Eastern Region of Malaysia. The prevalence and the changing trend of the principal cause of blindness and VI could have been attributed to the KK-KKM, a cataract surgical outreach project operational at the community level which emphasized surgical outcomes as its service output. The project could further be utilized as a platform for multisectoral collaboration for community advocacy in cataract surgery and to address the high percentage of avoidable blindness within both regions.
