# Supplementary material for: Prevalence of visual impairment and its causes in adults aged 50 years and older: Estimates from the National Eye Surveys in Malaysia
Source: PLoS One. 2024 Oct 24;19(10):e0299768. doi: 10.1371/journal.pone.0299768 (PMC11500914; doi:10.1371/journal.pone.0299768)
Supplement: Attachment — Submitted filename: Response_to_Editor_PLOS ONE Journal II.doc [file pone.0299768.s001.doc]

PLOS ONE Journal

Manuscript Title: Prevalence of visual impairment and its causes in adults aged 50 years and older: Estimates from the National Eye Surveys in Malaysia

Dear Jonafer Estoque Miranda,

I am writing to inform you that we have taken note of your feedback regarding the ethics approval letter for our study. We apologize for the oversight in not including the entire date range for participant recruitment (from 27 July 2023 to 7 October 2023) in the initial ethics approval letter. We understand the importance of ensuring that all necessary documentation is in place to maintain the integrity of our research.

In order to rectify the issue, we have compiled and uploaded all the ethics approval documents that cover the entire study process period from 2019 to 2024. We have carefully reviewed our records and have ensured that all the relevant ethics approval letters are now available for your reference. These documents provide a comprehensive overview of the ethical considerations and permissions granted for our study during the specified time frame.

Should you require any further information or have any additional concerns, please do not hesitate to reach out to us. We are committed to providing any necessary documentation or clarification to support the publication process.

Thank you for your understanding and cooperation. We greatly appreciate your guidance and look forward to your continued support throughout the publication process.

Thank you for your attention to this matter.

Kind regards,

Prof Dr Nyi Nyi Naing @ Syed Hatim Noor

Faculty of Medicine,

Universiti Sultan Zainal Abidin,

Terengganu, Malaysia

Corresponding Author

Phone no: +60179381610

Date:15 February 2024
