# Supplementary material for: Prevalence of visual impairment and its causes in adults aged 50 years and older: Estimates from the National Eye Surveys in Malaysia
Source: PLoS One. 2024 Oct 24;19(10):e0299768. doi: 10.1371/journal.pone.0299768 (PMC11500914; doi:10.1371/journal.pone.0299768)
Supplement: Attachment — Submitted filename: Response to Reviewers.doc [file pone.0299768.s003.doc]

**PLOS ONE Journal**

**Manuscript Tittle: Prevalence of visual impairment and its causes in adults aged 50 years and older: Estimates from the National Eye Surveys in Malaysia.**

Dear

Kumar Saurabh
Academic Editor
PLOS ONE

Thank you for considering our manuscript for publication in PLOS ONE. We appreciate the time and effort that the reviewers and editorial team have put into evaluating our submission. We have modified the manuscript accordingly and the detailed corrections are listed below point by point:

**Editor comments on journal requirements:

Please review your reference list to ensure that it is complete and correct. If you have cited papers that have been retracted, please include the rationale for doing so in the manuscript text, or remove these references and replace them with relevant current references. Any changes to the reference list should be mentioned in the rebuttal letter that accompanies your revised manuscript. If you need to cite a retracted article, indicate the article’s retracted status in the References list and also include a citation and full reference for the retraction notice.**

Response to editor:

**We believe that our reference lists are complete and correct, and none of them have been officially retracted.**

**Reviewer #2:**

**Comment**

**The authors adequately addressed the comments provided by this reviewer. Two minor considerations should be made:**

**1. Verification from the authors and editor that the manuscript does not exceed the word count requirement of the publication.**

Response to comment:

We have reviewed the manuscript and confirmed that it adheres to the journal's guidelines regarding word count, number of figures, and supporting information. As per the journal's instructions, there are no restrictions on these elements. We have ensured that the manuscript is presented in a concise and clear manner, while providing sufficient detail to support our findings.

**2. The explanatory sentence starting in line 186 can be removed. The comment arose from the notion that earlier versions of RAAB conventionally used pinhole as best corrected.**

[**https://www.raab.world/analysis/uncorrected-va-and-erec-raab**](https://www.raab.world/analysis/uncorrected-va-and-erec-raab)**.**

Response to comment:

We agree with the reviewer’s observation and have removed the explanatory sentence starting in line 186 from the revised manuscript.

Line 186

The best correction would require refraction, which would be time-consuming and impossible in the field without proper equipment and expertise. It was also not measured because it would deviate from RAAB protocol, which only measured corrected VA (using a pinhole).

**Reviewer #3:**

**Comment**

**I thank the authors for appropriately addressing all queries. I would also request that other national level eye surveys in the WHO SEAR region such as Nepal, India, Bhutan and Cambodia may be included in discussion apart from the Indonesian data. That will provide useful context to the situation in entire WHO-SEAR region.**

**Some relevant primary references are:**

1. **Lepcha NT, Sharma IP, Sapkota YD, Das T, Phuntsho T, Tenzin N, et al. Changing trends of blindness, visual impairment and cataract surgery in Bhutan: 2009-2018. PLoS ONE 2019;14(5):e0216398.**
2. **Mishra SK, Shah R, Gogate P, Sapkota YD, Gurung R, Shrestha MK, et al. Prevalence of blindness and vision impairment among people 50 years and older in Nepal: a national Rapid Assessment of Avoidable Blindness survey [Internet]. 2024 [cited 2024 Aug 11]; Available from:** [**http://medrxiv.org/lookup/doi/10.1101/2024.08.06.24311588**](http://medrxiv.org/lookup/doi/10.1101/2024.08.06.24311588)
3. **Vashist P, Senjam SS, Gupta V, Gupta N, Shamanna BR, Wadhwani M, et al. Blindness and visual impairment and their causes in India: Results of a nationally representative survey. PLoS One 2022;17(7):e0271736.**
4. **Cambodia 2019 -** [**https://www.raab.world/survey/cambodia-2019**](https://www.raab.world/survey/cambodia-2019)

Response to comment:

Thank you for your valuable feedback and for highlighting the importance of a broader regional context. We have incorporated data from these countries into our discussion to provide a more holistic view of the eye health landscape within WHO-SEAR, highlighted in RED font. (Line 307-310)

Line 307-310

Malaysia’s blindness prevalence was relatively comparable to the South East Asia Region (SEAR) contexts, indicating similar eye health challenges in the region (Nepal 1.1%, Bhutan 1.0%, India 2.0% and Cambodia 2.5%) [22-25].

**References**

22. Lepcha NT, Sharma IP, Sapkota YD, Das T, Phuntsho T, Tenzin N, et al. Changing Trends of	Blindness, Visual Impairment and Cataract Surgery in Bhutan: 2009-2018. *PLoS One.*	2019;14(5):e0216398. DOI: 10.1371/journal.pone.0216398 PMID: **31071127**
23. Mishra SK, Shah R, Gogate P, Sapkota YD, Gurung R, Shrestha MK, et al. Prevalence of Blindness and Vision Impairment Among People 50 years and Older in Nepal: A National Rapid Assessment of Avoidable Blindness Survey [Internet]. 2024 [cited 2024 Aug 11];Available from: <http://medrxiv.org/lookup/doi/10.1101/2024.08.06.24311588>. DOI: <https://doi.org/10.1101/2024.08.06.24311588>
24. Vashist P, Senjam SS, Gupta V, Gupta N, Shamanna BR, Wadhwani M, et al. Blindness and Visual Impairment and Their Causes in India: Results of A Nationally Representative Survey. *PLoS One.* 2022;17(7):e0271736. DOI: [10.1371/journal.pone.0271736](https://doi.org/10.1371/journal.pone.0271736) PMID: [35862402](https://pubmed.ncbi.nlm.nih.gov/35862402)
25. RAAB.(2019). Cambodia 2019 Survey Data. Retrieved from [https: //www.raab.world/survey/cambodia-2019](https://www.raab.world/survey/cambodia-2019)
